# A delayed presentation of traumatic diaphragmatic hernia in a young male: a unusual case report and comprehensive review of literature

**DOI:** 10.1093/jscr/rjae613

**Published:** 2024-10-02

**Authors:** Suraj K C, Samiksha Lamichhane, Bhawani Khanal, Abhijeet Kumar, Rakesh Kumar Gupta

**Affiliations:** Department of General Surgery, BP Koirala Institute of Health Sciences, Buddha Road, Dharan, Sunsari, 56700, Nepal; Department of Radiodiagnosis and Imaging, BP Koirala Institute of Health Sciences, Buddha Road, Dharan, Sunsari, 56700, Nepal; Department of General Surgery, BP Koirala Institute of Health Sciences, Buddha Road, Dharan, Sunsari, 56700, Nepal; Department of General Surgery, BP Koirala Institute of Health Sciences, Buddha Road, Dharan, Sunsari, 56700, Nepal; Department of General Surgery, BP Koirala Institute of Health Sciences, Buddha Road, Dharan, Sunsari, 56700, Nepal

**Keywords:** diaphragmatic hernia, incarceration, perforation, mesh

## Abstract

Acquired diaphragmatic hernia is typically caused by blunt trauma to the abdomen. It can be challenging to diagnose in acute cases due to a wide range of symptoms. Delayed presentation of traumatic diaphragmatic hernia is uncommon and can lead to respiratory issues or bowel complications like incarceration, perforation, or strangulation. Computed tomography is the preferred diagnostic tool. For acute case, laparotomy is indicated traditionally; however, the choice of surgery is dependent upon the surgeon’s expertise and availability of resources.

## Introduction

A defect within the diaphragm causes the protrusion of abdominal contents into the thoracic cavity, known as diaphragmatic hernia. It is most commonly a congenital phenomenon; however, there have also been cases where it can be acquired [1]. Congenital causes of diaphragmatic hernia should be considered in all cases if trauma has not occurred prior to the presentation [2]. Acquired diaphragmatic hernia is secondary to trauma to the diaphragm, mostly occurring at potential areas of weakness as seen in embryological fusion points. The main pathophysiology behind traumatic hernia is a sudden increase in the pleuroperitoneal pressure gradient [3]. A traumatic diaphragmatic hernia may be misdiagnosed acutely due to its nonspecific clinical features and radiological findings, often presenting months or years after the trauma [4]. Our case involves a 28-year-old male who sustained a road traffic accident one year prior and developed abdominal distension, abdominal pain, and multiple episodes of nonbilious vomiting. He was found to have a left-sided diaphragmatic hernia with bowel loops as content. He was managed with laproscopic diaphragmatic hernia repair with composite mesh hernioplasty.

## Case presentation

A 28-year-old male presented to the emergency room with acute epigastric and left hypochondrium pain for 3 days. He complained of not passing stool and flatus for the same duration, with multiple episodes of non-bilious, non-projectile vomiting. He gave no history of abdominal distension, fever, or shortness of breath. He gives a history of a road traffic accident 2 years ago with impact over left chest and rib fractures, for which a thoracostomy tube was placed; however, documents are not available currently. Examination findings showed tachypnea with a respiratory rate of 22 cycles per minute. The abdomen was soft and non-distended, with tenderness in the left hypochondrium and epigastric region. Respiratory examination revealed the absence of breath sounds at the lower chest with audible bowel sounds. Other examinations were normal.

**Figure 1:**
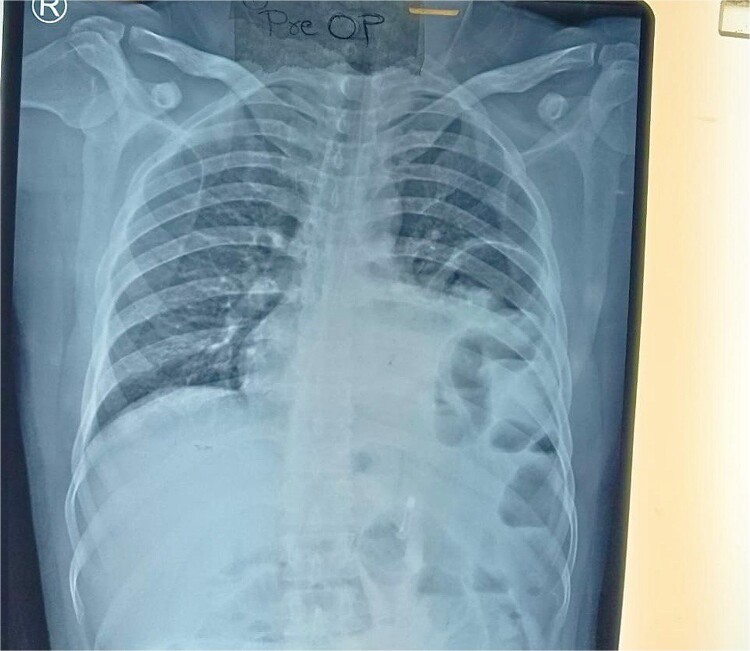
Chest X-ray PA view showing the herniation of bowel loops into the left hemithorax with collapse of the lower zone of the lung.

**Figure 2:**
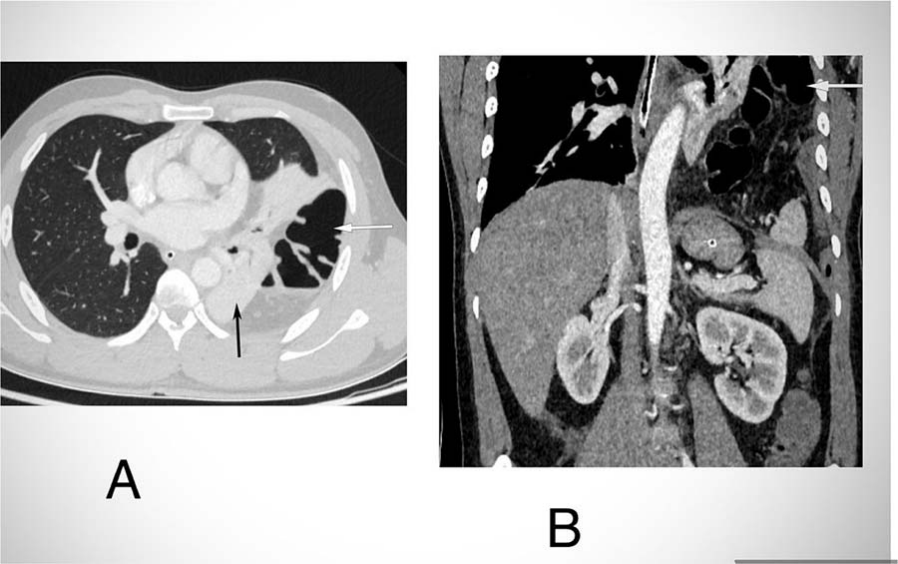
CECT abdomen and lower chest in axial section in lung window (A) and coronal section (B) shows herniation of transverse colon and its mesocolon (white arrow) through a defect in left hemidiaphragm into the hemithorax. There is passive atelectasis of the lower lobe of the left lung (black arrow in A).

**Figure 3:**
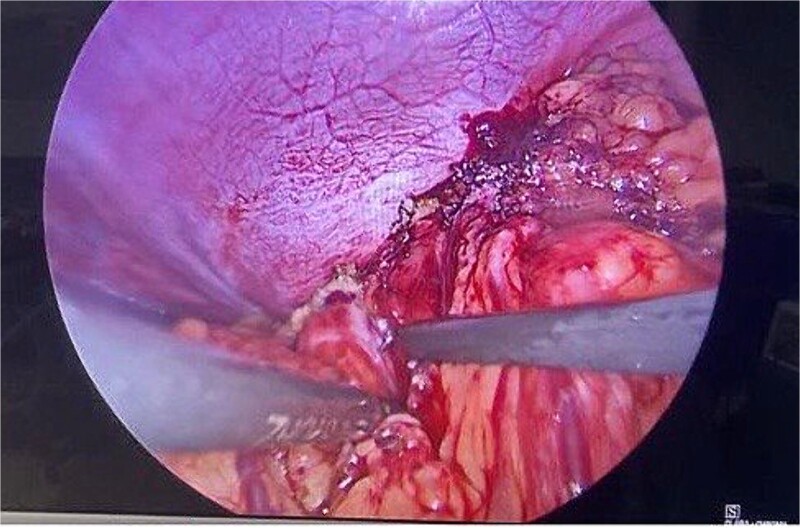
Intraoperative picture showing the defect in the left diaphragm with the omentum and bowel loops as content.

**Figure 4:**
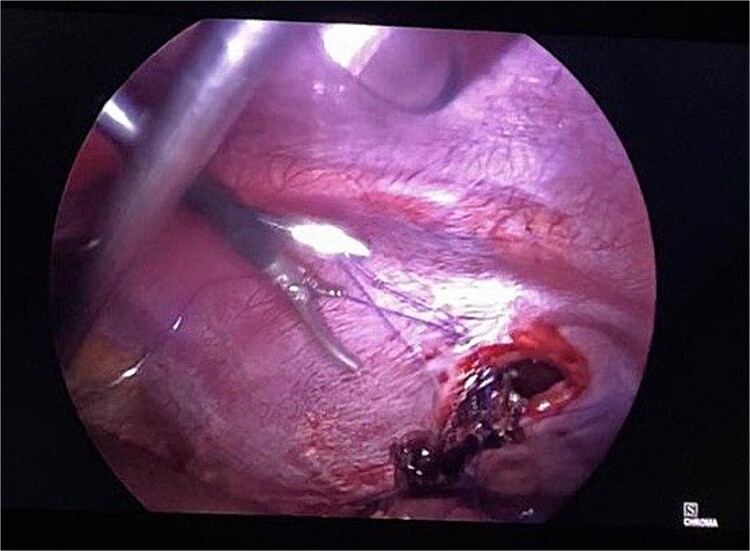
Intraoperative picture following reduction of hernia. The defect is being closed with prolene.

**Figure 5:**
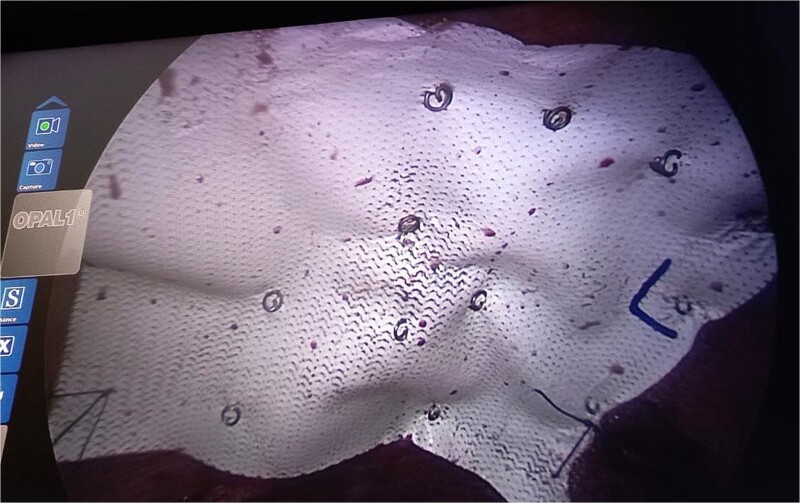
Intraoperative picture showing composite mesh placement over the defect.

**Figure 6:**
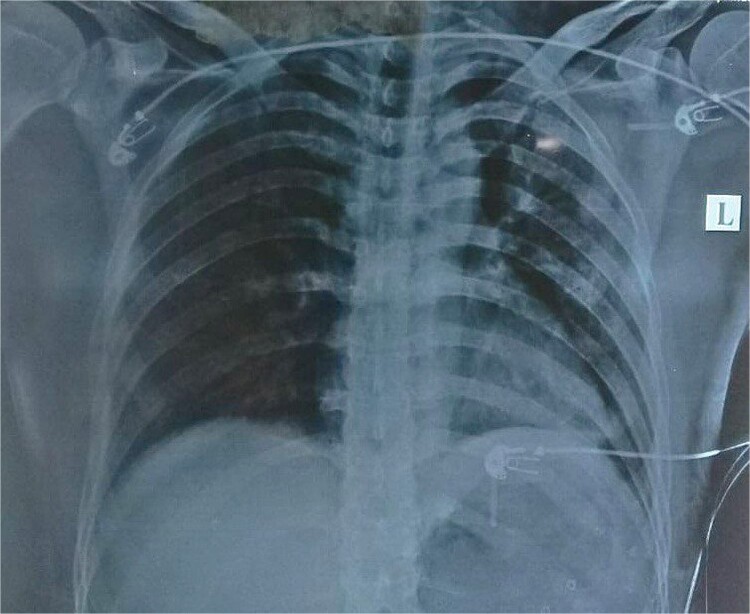
Post-operative picture with no evidence of herniation of bowel content to the left hemidiaphragm.

Routine examinations, including total blood counts, differential blood counts, renal function tests, and liver function tests, were within normal limits. The chest X-ray PA view showed the herniation of bowel loops into the left hemithorax with the collapse of the lower zone of the lung with no evidence of previous fracture (Fig. 1). Computed tomography (CT) showed a defect of size around 4 cm with evidence of protrusion of intra-abdominal content in the left hemidiaphragm with basal atelectasis of the left lung and mild dilatation of the jejunal bowel loops with a few air-fluid levels without a transition point (Fig. 2). The patient underwent laparoscopic left diaphragmatic hernia repair with mesh hernioplasty after 6 h of admission. The intraoperative findings revealed a defect of size 4 cm × 2 cm at the posterolateral aspect of the left hemidiaphragm with protrusion of the transverse colon, omentum, and part of the spleen with adhesions (Fig. 3). The content of the hernia was reduced, and primary repair of the defect was done (Fig. 4), and the defect was closed with a 10 × 15 cm intraperitoneal onlay composite mesh (Fig. 5). A postoperative X-ray showed no evidence of herniation of the bowel loops above the left hemidiaphragm (Fig. 6). He was discharged on the fourth postoperative day with oral medications. Follow-up in the outpatient department on the 14th postoperative day showed that he was doing well.

## Discussion

Blunt diaphragmatic rupture is most commonly seen in road traffic accidents, as evidenced in our case. The incidence of post-traumatic blunt diaphragmatic injury is rare and ranges from 0.16% to 5%, according to various literature reviews [4]. The left hemidiaphragm is commonly injured due to an area of congenital weakness in its posterolateral aspect. Diaphragm ruptures occur ~10 times more frequently on the left side in cases of blunt trauma due to the fact that the left medial and posterolateral sections of the diaphragm are less robust during embryonic development. The liver acts as a protective barrier on the right side, which reduces the incidence of ruptures [5]. The possible mechanisms resulting in traumatic diaphragmatic hernia include anteroposterior elongation and shearing of the diaphragm in lateral thoracoabdominal impact [6]. Up to 70% of diaphragmatic tears are initially missed [7], and 7.2% of injuries that are missed in the acute stage end up with complications. The herniation of abdominal contents and viscera through the defect may lead to compromise [6]. The patient with diaphragmatic hernia presents with marked respiratory distress, decreased breath sounds on the affected side, and auscultation of bowel sounds in the chest. One can appreciate paradoxical movements of the abdomen with breathing, diffuse abdominal pain, chest pain, and vomiting [8]. Abdominal symptoms may include recurrent abdominal pain, postprandial fullness, vomiting, and obstructive gastrointestinal symptoms [3]. Diaphragmatic hernias can be divided into three types: Type 1 hernia is diagnosed immediately following trauma; Type 2 hernia is diagnosed within the recovery period of trauma; and Type 3 hernia is diagnosed when the patient presents with ischemia or perforation of herniated organs and is diagnosed late [9]. The patient usually has a scaphoid abdomen with audible bowel sounds and an absence of breath sounds in the lower chest area, which was consistent with our case. Radiological imaging is essential for the diagnosis of hernia and includes chest radiographs, ultrasonography, magnetic resonance imaging, and CT. CT is the modality of choice [10], with a sensitivity and specificity of 61%–87% and 72%–100%, respectively [11]. Acute traumatic diaphragmatic hernia is managed with resuscitation followed by surgical correction [12]. Surgery is the preferred method of treatment for diaphragmatic hernia and can be performed through various approaches [13]. Laparotomy or thoracotomy are the traditional treatment approaches, the choice being largely dependent on the skill set of the surgeon involved. The advent of laparoscopy has provided a new approach to this clinical situation. However, reports of laparoscopic or laparoscopic-assisted hernia repair are scarce and are generally limited to chronic post-traumatic or congenital hernias [14]. When primary closure with non-absorbable sutures is not possible due to a large defect size, mesh repair may be an alternative. We have used a composite mesh of size 10 × 12 cm to cover the defect. The thoracotomy or combined thoracic-abdominal approach is generally preferred to reduce viscera-pleural adhesions and intra-thoracic visceral perforation in cases of delayed presentation [3]. Minimally invasive laparoscopic or thoracoscopic approaches are preferred due to fewer complications [15]. The work is done as per the SCARE 2023 criteria [16].

## Conclusion

Blunt trauma to the abdomen may result in traumatic diaphragmatic hernia. In the acute setting, hernias are often misdiagnosed due to vague presentations. Symptoms may include a scaphoid abdomen, decreased breath sounds in the lower lung field, and audible bowel sounds. CT is the preferred diagnostic tool. Surgery is the preferred treatment for diaphragmatic hernias. Laparoscopy, although reducing mortality and morbidity, can be challenging in long-standing hernias due to incarceration and adhesions.

## Consent

Written consent was obtained from patient.
